# Old Myths, New Concerns: the Long-Term Effects of Ascending Aorta Replacement with Dacron Grafts. Not All That Glitters Is Gold

**DOI:** 10.1007/s12265-016-9699-8

**Published:** 2016-05-31

**Authors:** Cristiano Spadaccio, Francesco Nappi, Nawwar Al-Attar, Fraser W. Sutherland, Christophe Acar, Antonio Nenna, Marcella Trombetta, Massimo Chello, Alberto Rainer

**Affiliations:** 1 Department of Cardiothoracic Surgery, Golden Jubilee National Hospital, Agamemnon Street Clydebank, Glasgow, G81 4DY UK; 2 College of Medical, Veterinary and Life Sciences, Institute of Cardiovascular and Medical Sciences, University of Glasgow, Glasgow, UK; 3 Cardiac Surgery Centre Cardiologique du Nord de Saint-Denis, Paris, France; 4 Department of Cardiothoracic Surgery, Hôpital Pitié-Salpétrière, Paris, France; 5 Department of Cardiovascular Sciences|, University Campus Bio-Medico of Rome, Roma, Italy; 6 CIR—Laboratory of Tissue Engineering, Università Campus Bio-Medico di Roma, Roma, Italy

**Keywords:** Aortic compliance, Windkessel, Hemodynamics, Dacron, Aortic replacement, Aortic surgery, Aortic root, Complications

## Abstract

Synthetic grafts are widely used in cardiac and vascular surgery since the mid-1970s. Despite their general good performance, inability of mimicking the elastomechanical characteristics of the native arterial tissue, and the consequent lack of adequate compliance, leads to a cascade of hemodynamic and biological alterations deeply affecting cardiovascular homeostasis. Those concerns have been reconsidered in more contemporaneous surgical and experimental reports which also triggered some research efforts in the tissue engineering field towards the realization of biomimetic arterial surrogates. The present review focuses on the significance of the “compliance mismatch” phenomenon occurring after aortic root or ascending aorta replacement with prosthetic grafts and discusses the clinical reflexes of this state of tissue incompatibility, as the loss of the native elastomechanical properties of the aorta can translate into detrimental effects on the normal efficiency of the aortic root complex with impact in the long-term results of patients undergoing aortic replacement.

## Introduction

Synthetic grafts, including expanded poly(ethylene terephthalate) (Dacron®) or poly(tetrafluoroethylene) (ePTFE), have been available since the mid-1970s and represent the most widely employed vascular substitutes in cardiovascular surgery. Their large availability and ease of use have allowed an extensive application of these conduits in a wide spectrum of vascular pathologies including aneurysms of large vessels and atherosclerosis of peripheral districts.

The concept of “compliance mismatch” between the graft and native aorta has been introduced to explain what emerged as a silent but worrying concern regarding the reflexes that the presence of prosthetic materials can determine on the native cardiovascular structures and consequently on the long-term outcomes of these artificial grafts [1]. After 50 years since the initial implantation of Dacron grafts and despite their general good performance, dilation or aneurysm, para-anastomotic pseudoaneurysm, and mechanical failure are still considered daunting issues in the follow-up of these patients often requiring additional surgery [2, 3]. Several early studies revealed that a common pathogenic mechanism underlying the failure of these conduits was represented by the mismatch between the actual biomechanical properties of the grafts and those of the native vascular tissue [4]. Indeed, current available vascular grafts exhibit more than four times reduced compliance in respect to native arteries (1.8 × 10^−2 %^ versus 8 × 10^−2 %^ mmHg^−1^) [4]. Inability of mimicking the elastomechanical characteristics of the native arterial tissue, and the consequent lack of adequate compliance of the grafts, leads to dilation with subsequent flow anomalies and is able to trigger a perpetuating circle of vascular wall alterations causing detrimental reflexes both locally and systemically [1]. Furthermore, insertion of synthetic material into the arterial system was also shown to reflect in endothelial dysfunction and thrombosis [5–8]. Indeed, the lower mechanical compliance generates a disturbed flow pattern promoting neo-intimal hyperplasia especially at anastomosis site [5, 9].

However, some other studies pointed out that the presence of an inextensible segment within the vascular tree, especially at the level of the aorta ascendens, could determine also local suture overstress, leading to several prosthesis-related complications, but, more importantly, could ultimately exert retrograde deleterious effects on valve competence, cardiac function, and perfusion [1].

Those early reports were already pointing at these potential long-term complications related to the use of prosthetic materials in the vascular tree but have been unjustly neglected in front of the clinical success of the implants. However, the recent renovated interest of the bioengineering science in the development of biomimetic compliant prosthesis [5, 10] together with the pessimism expressed by some in the surgical literature regarding the long-term performance of Dacron or similar materials [11, 12] intriguingly reflect the current perception of some negative aspects in the use of artificial materials in the arterial system which are susceptible of improvement. Thus, we re-collected these evidences and reviewed the literature focusing on the significance of the compliance mismatch phenomenon occurring after aortic root or ascending aorta replacement with prosthetic grafts and we compared that with the current knowledge. The clinical reflexes of this state of “tissue incompatibility” are taken in consideration to underline that the loss of the native elastomechanical properties of the aorta can translate into detrimental effects on the normal efficiency of the aortic root complex and might pose daunting concerns in the long-term results and clinical management of aortic replacement in the clinical practice.

## Methods

PubMed, EMBASE, and Cochrane Library database were searched for review, metaanalysis, and original articles inserting as key words “aortic,” “Dacron,” “compliance,” “complication,” and “long term.” Search was restricted to cardiac surgery, excluding vascular or interventional radiology studies. Full text was obtained for the majority of the studies with the exception of articles not published in English. In these cases, only abstracts were considered.

## Results and Discussion

The long-term effects of ascending aorta replacement with synthetic Dacron graft might be collectively classified as antegrade, mainly regarding issues at the prosthesis site, and retrograde, which entail to the hemodynamic reflexes of the compliance mismatch on the left ventricle and aortic valve.

### Antegrade Effects

Structural constraints, intrinsic mechanical properties of Dacron grafts and physical changes occurring after their implantation, are at the root of the inadequate elastic response of these prostheses and give rise to a compliance mismatch phenomenon that translates in a cascade of functional problems, eventually reaching the clinical scenario. Indeed, disadvantageous compliance properties and thrombogenicity especially at the anastomosis site have been claimed to be the two as the two major causes of the early and late graft failure [5].

**Fig. 1:**
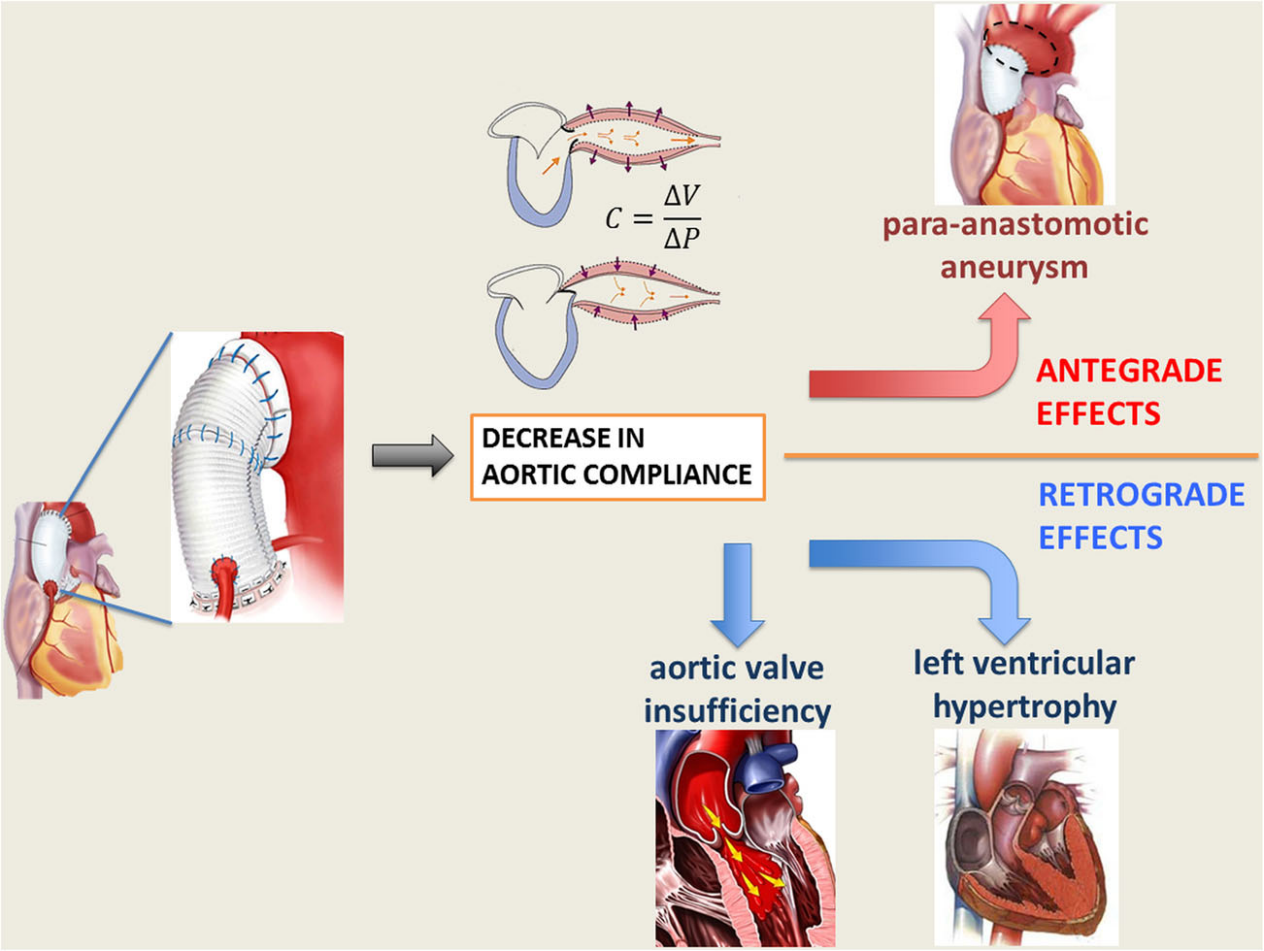
Schematic representation of antegrade and retrograde effects of ascending aorta replacement with non-compliant prosthetic graft

The unfavorable elastomechanical properties and the compliance mismatch between the native aorta and the Dacron graft are thought to impart excessive stress at the suture lines resulting in intimal hyperplasia, anastomotic aneurysms, or pseudoaneurysm [13, 14] (Fig. 1). In the clinical reality, pseudoaneurysm after ascending aorta replacement represents a daunting issue, generally determined by a total or partial dehiscence of the prosthesis from the aortic wall [15]. Region of contact between artificial materials (i.e., Prolene^™^ suture and Dacron) and aorta, together with the inflammatory tissue elicited by the foreign body presence, are considered a *locus minoris resistentiae* and are usually at the basis of this complication. Pseudoaneurysm can appear as a frank increase in diameter; compression of adjacent organs; or as occurrence of life-threatening complications such as rupture, fistula formation, or thrombosis [15]. Current literature reports an incidence of pseudoaneurysm after aortic dissection repair from 2 up to 6 % [16–18]. Replacement with homograft or simple repair with direct suture seems to be the elective treatment for this complication, though characterized by high morbidity and mortality, which ranges between 12 and 41 % [19, 20].

In the pediatric population, one of the most daunting long-term complications of prosthetic aorta replacement regards the development of false aneurysm of the suture line. Troost et al. reported an 8 % incidence in the long-term follow-up and interestingly noted as direct surgical repair of aortic coarctation without graft interposition was not associated with pseudoaneurysm, claiming that a surgical suture problem or continuous traction on the suture lines on inextensible materials would be potentially responsible for this issue [21].

Additional examples of this tissue incompatibility derive again from pulmonary autograft reinforcement with Dacron prosthesis during Ross operation and from experimental findings demonstrating strong inflammatory responses triggered by prosthetic materials when used to wrap the exterior surface of arterial vessels [22]. Abnormal and unregulated foreign body reaction might severely impair tissue growth and elastic remodeling and further hemodynamic function of the neo-aorta [23].

Another well-documented event is represented by the long-term size change of Dacron grafts used in the ascending aorta. A study from Takami et al. [24] demonstrated a 26 % diameter increase, measured immediately after implantation, compared to the package size, a successive dilation of 10.5 % versus the diameter at discharge, and an increase of 3.23 % per year over a 5-year follow-up period. Beside the caveat related to the fact that this evaluation was performed in thoracic descending aorta, with a known difference in hemodynamics compared to ascending aorta, the results are in agreement with previous data published by Mattens et al. reporting a 31.4 % dilation over 2-year follow-up with a similar per-year rate of increase [25]. Early studies from Berger et al. [26] showed alterations in the microstructure of Dacron grafts that may translate into different degrees of graft dilatation [2] or rarely determine graft rupture due to material fatigue [27, 28]. However, the physical changes in the yarn architecture together with the in vivo material degradation in contact with biological fluids determine loss of elasticity and change in compliance of the graft that is further responsible of the dilation profile shown by these prostheses [29, 30] and their functional consequences [1].

### Retrograde Effects

The lack of adequate elastomechanical properties leads to deleterious effects not only at the graft site but also retrogradely with significant reflexes on cardiac function and aortic valve competence (as schematized in Fig. 1). The introduction of a non-distensible graft and the compliance mismatch with the highly elastic native aorta have been shown to determine a significant change in the aortic vascular properties, eventually resulting in increased ventricular afterload [31, 32]. Characteristic impedance and pulse wave reflection, parameters describing vascular compliance, are dramatically affected, and the loss of Windkessel effect and alteration of the pulse wave propagation translate in additional workload for the left ventricle eventually inducing adaptive hypertrophy [32, 33]. The finding of increased aortic stiffness and augmented aortic pulsed wave velocity (PWV) have been widely reported in the elderly population in which increased aortic rigidity translates in ventricular hypertrophy and reduced coronary flow because of the loss of diastolic augmentation [34]. Increases of 1 m/s of PWV has been found to increase cardiovascular risk by 15 % in a recent metaanalysis [35], and Kidher et al. inserted this parameter in the cardiac surgery context reporting a significant association between increased PWV of native aorta and postoperative NYHA status after aortic valve replacement in the elderly [36]. A clinical study comparing age-matched subject bearing a thoracic aortic graft with healthy controls demonstrated under exercise a more marked increase in aortic impedance and an excessive reduction in pulse wave reflection in respect to controls, indicating an higher aorta-graft compliance mismatch when high output flow is required [33]. In these circumstances, the Dacron graft behaved as a functional stenosis, generating a significant change in impedance at the interface with the native elastic artery and secondarily determining a reduction in ventricular pumping efficiency. The latter was associated to a higher cardiac energetic cost to maintain a given flow in the less compliant arterial system [33]. Interestingly, clinical reports of patients who had undergone ascending and/or abdominal aorta bypass for thoracoabdominal aneurysm using woven Dacron grafts demonstrated the development of left ventricle hypertrophy [37, 38]. Further studies documented similar consequences after proximal or long bypass procedures with non-compliant grafts, identifying increased characteristic impedance, decreased Windkessel effect of the proximal aorta, and increased systolic wall stress as the main potential factors [32, 38–40]. More importantly, Kass et al. [41] demonstrated that vasculature stiffening induces cardiac dysfunction and ischemia for increased oxygen demand and alteration in phasic coronary flow. Also, the group of Toutouzas et al. remarked the importance of aortic elastic properties as determinants of both ventricular function and coronary flow [42], and aortic distensibility was shown to positively influence myocardial ischemic preconditioning [43]. Translation of these data into the clinical scenario meets the evidence of a natural decrease of arterial compliance with age, with a parallel increase in systolic pressure [44]. This aspect might further complicate the hemodynamic situation especially considering that aortic reconstructive surgery is often performed on elderly patients, whose aortas are already further up in the degenerative process and might have reduced compliance. Additionally, increased aortic stiffness has been associated to left ventricular diastolic dysfunction in hypertensive patients [45]. Introduction of non-compliant grafts in these circumstances, with the consequent further reduction in compliance, might lead to even more relevant hemodynamic changes. Interestingly, one of the first studies demonstrating hemodynamic and ventricular changes in patients undergoing thoracic aorta replacement with prosthetic grafts included a series of subjects with ages ranging between 48 and 60 years [37]. Therefore, these consequences might not be imputed to original abnormal or deteriorated conditions of the native aorta. Data on the long-term hemodynamic outcomes in even older patients could further elucidate this phenomenon and might be interesting in order to predict eventual outcomes in aged population.

The consequences of the compliance mismatch on the aortic valve function might be even more striking in the clinical scenario. Loss of compliance and augmented stiffness of aortic root determine and increased load of aortic valve cusps, leading to their dysfunction and accelerating their degeneration [12, 46]. Interestingly, David et al. [47] have recently published results on a large series of aortic root replacements using the re-implantation technique, which implies the use of Dacron tubular prostheses without the remodeling of neo-aortic sinuses. These authors showed an acceptable long-term outcome in terms of postoperative aortic insufficiency. The freedom from reoperation on the aortic valve at 1, 5, 10, and 15 years were 99.7 ± 2.0, 99.7 ± 2.0, 97.8 ± 5.3, and 97.8 ± 5.3 %, respectively [47], and similar data were reported in another series [48]. Reoperation is usually performed for severe aortic insufficiency, but careful analysis of the echocardiographic follow-up data of the operated patients throughout different studies indicates that a cumulative percentage of more than 40 % patients showed a mild-to-moderate aortic valve insufficiency [49], while actual freedom from severe insufficiency at 1, 5, 10, and 15 years were 99.6 ± 0.8, 98.3 ± 3.5, 92.9 ± 6.5, and 89.4 ± 12 %, respectively, according to David et al. [47]. Therefore, despite the degree of aortic valve dysfunction does not achieve a surgical significance or mandate for reoperation, these data represent an evidence that prosthetic replacement of aortic root might influence the mechanical properties of the aorta and the hemodynamic parameters of the aorta-valve complex, inducing valve malfunctioning. The above-cited studies presented a mean follow-up of 60 months, showing acceptable performance, but it is still unclear whether the aortic insufficiency induced by the rearrangement of the aortic root or by the simple valve degeneration is able to progress in 10 years up to a level requiring reintervention. However, as stated by David et al., “surgery does not arrest the degenerative process and aortic valve function may deteriorate with time.” Considering the young age of the patients usually treated with aortic valve-sparing techniques, these strategies might represent the best option when permanent oral anti-coagulation is unwanted or non-practicable, but “patients need to be aware of the possible necessity of reoperation in the future” [47]. However, despite that it is difficult to establish a causative effect between the reduced compliance of the neo-aortic root and the general degenerative evolution of the aortic valve, it has been postulated that a rigid aortic root may accelerate degenerative changes in the aortic cusps [50]. In this context, Zehr et al. [51], in their report on 30-year experience in aortic root replacement, pointed out that the vicinity of the Dacron tube to the cusps can result in trauma to the leaflets when they open hitting the tube graft. The hemodynamic load exerted on an inextensible graft might also reflect in a progressive annular dilation eventually leading to recurrent aortic insufficiency, and this effect might be more pronounced in Marfan syndrome patients, in whom congenital abnormal fibrillin-1 metabolism can render residual aortic tissues even weaker and more prone to dilation [51]. These points have been stressed by Rama and colleagues in 2007 when, taking from the incidence of annulus dilatation and cusp damage and dysfunction, they suggested a new technique of valve-sparing aortic root replacement aiming at preserving and reconstituting anatomical and geometrical features of sinotubular junction and preventing traumatic damage of cusps against inextensible Dacron prosthesis [52]. Therefore, another important element to be considered in the definition of aortic root compliance and of the dynamics of the valve-aortic root complex is represented by the Valsalva sinuses. A large piece of experimental effort has been spent since the first historical description of Bellhouse and Bellhouse in the early 1970s, which demonstrated the dynamic function of sinuses in the modulation of aortic valve closure [53]. The majority of in vitro and the in vivo studies, in both native or surgical reconstructed aortic roots, are pointing at the role of the sinuses during the diastolic phase in positively regulating the leaflet dynamics during the cardiac cycle [54, 55] and in coronary flow modulation [56]. The hydrodynamic properties of the aortic root provided by the presence of the sinuses allow for reduced transvalvular gradient and leaflet motion, while their absence reduces the time required for leaflet coaptation, increases the valve closing volume and the maximum transvalvular flow velocity, determining an increase in the working stress on the valve tissue, with eventual premature structural valve deterioration [57]. In fact, the normal compliance of the root and its sinuses is considered at the basis of the normal leaflet dynamics. The systolic expansion of the aortic root helps the free margins of the leaflet to maintain a distended and flat configuration during opening. Loss of root compliance might determine free margin folding and wrinkling because of reduced excursion from the close to total opening position, eventually leading to accelerated degeneration with valve dysfunction [58]. As recognized by the same inventor of the reimplantation technique, when reconstructing the aortic root with inextensible anelastic Dacron conduit, leaflet motion will occur inside a stiff and rigid prosthesis with increased risk of valve dysfunction and early degeneration [59]. The importance of recreate neo-sinuses during the procedure has been discussed and also testified by the last modification of David operation which includes sinotubular ridge reconstruction performed by adapting the graft in the spaces in between commissures in order to resemble pseudosinuses [59, 60]. Indeed, the use of a straight Dacron graft abolishes the physiological geometry of the sinuses and sinotubular junction and it has been demonstrated that the velocity of opening and closure of the aortic cusps is greatly increased in this operation [61], but it can be decreased by creating neo-aortic sinuses [62] or by using the Valsalva graft [63]. To this extent, an interesting in vitro dynamic study by Pisani and colleagues, simulating the surgical situation of implantation of stentless Sorin Solo^™^ valve with or without the reconstruction of neo-sinuses, demonstrated that the presence of Valsalva sinuses is crucial not only in the diastolic phase but also in the systolic phase and when the cardiac output increases. Simulating an increase of cardiac performance, as per physical exercise, these authors demonstrated that the presence of the sinuses ensured an increase in the valve effective orifice area guaranteeing to maintain the transvalvular gradient unchanged for each increase in the cardiac output. Therefore, preservation of aortic root compliance and adequate shape and dimension of Valsalva sinuses is crucial for the optimal and durable function of the aortic valve as the first allows a normal leaflet dynamics and motion with wrinkle-free cusp opening, while the latter ensures an efficient hemodynamic answer in conditions of increased cardiac output [64]. This finding acquires particular significance in the clinical scenario, as procedures of valve-sparing aortic root replacement or stentless valve full root implantation are usually performed in young active patients which might undergo important variations in the cardiac performance. To this extent, a clinical study comparing the hemodynamics in patients, who had undergone aortic root replacement using stentless valves with straight xenopericardial conduits or prosthetic Valsalva grafts, confirmed the importance of the elastic properties of the aortic conduit and demonstrated that the presence of neo-sinuses might improve the compliance of the aortic root determining a more physiologic flow pattern, as indicated by the maximum flow velocity above the aortic valve [65]. These findings stress the reported importance to use prosthetic conduits provided with neo-sinuses but also induce to consider the compliance of the graft itself for an optimal long-term result at the level of the aortic valve.

### Clinical Implications

Despite the overall successful performance of prosthetic Dacron graft in aortic surgery, an increasing amount of evidence is pointing at the discrepancy between the elastomechanical properties of the grafts and of the native aorta as the responsible of worrisome sequelae both locally, at the anastomotic site, and retrogradely, in term of valve dysfunction and ventricular workload.

Concerns on the lack of elasticity of Dacron grafts have been expressed also in the surgical literature especially in regards of their use to reconstruct hemodynamically important structures as Valsalva sinuses because of the progressive loss of compliance in vivo during encapsulation by fibrous tissue [11, 12]. The mismatch between prosthetic material and native aorta biomechanical properties is therefore more troublesome than initially thought, and prolonging this state of “tissue incompatibility” can exert detrimental effects on the normal efficiency of the aortic root complex. The loss of compliance induced by the interposition of an inextensible graft at the level of the aortic root not only might impart excessive stress on the suture line, leading to dangerous anastomotic aneurysm, but also blunt the favorable Windkessel function and hydrodynamic action provided by the root elastic expansion and Valsalva sinuses. Replacing or bypassing the aorta with non-compliant synthetic prostheses leads to serious changes in arterial wall, aortic leaflets, and ventricular loads [1], resulting in medium-term functional deterioration and in the need for reintervention in a small percentage of the population [66]. The optimal function of aortic valve following an aortic root replacement is not primarily determined by the presence and the geometry of Valsalva sinuses, but it is also deeply affected by the neo-root compliance [52, 62, 64]. Full root replacement with Valsalva Dacron conduits might not be sufficient to guarantee an adequate and efficient valve function in the long term because of the presence of an inextensible graft substituting the native aorta [11, 51]. Whether these alterations might acquire a stronger clinical significance in the long term, with an increase in the reoperation rate, cannot be deduced yet; longer-term follow-up studies are required for this purpose.

The ideal long-term solution would be to replace the native aorta with biological tissues such as xenografts/allografts or biomimetic prostheses obtained using tissue engineering approaches.

In this extent, it might be hypothesized that full root replacement using the currently available stentless porcine xenografts provided with the native sinuses and ascending aorta might partially preserve the normal compliance of aortic tissue and could be more appropriate for these purposes. However, the anti-antigenic and anti-calcification chemical treatments used in the preparation of these grafts might be detrimental on the compliance module of the prosthesis itself. Alternatively, new solutions deriving from the rapidly evolving world of regenerative medicine and bioengineering of tissues might be explored at least at the preclinical and translational levels.

### The Tissue Engineering Frontiers

With the aim to overcome the current shortcomings of artificial conduits, a significant amount of research has been lavished on the bioengineering front to generate biocompatible grafts able to guarantee an efficient endothelialization and maintenance of vascular patency [67]. However, in the recent years, scientific efforts have been centered on the realization of biomimetic grafts capable of reproducing when implanted the same hemodynamic and mechanical characteristics of the vascular tree [68]. Strategies employing dense fibrillar collagen networks combined with elastin-like proteins exhibited potential of realization of scaffolds endowed with an ultimate tensile strength (UTS) and Young’s modulus comparable to native vessels and showed better hemodynamic performance when compared to Dacron or expanded polytetrafluoroethylene [69]. Similarly, the combination of type I collagen gel and silk fibroin provided conduits with physiologically relevant compliance and resistance and improved the early response of the material to in vitro cell adhesion and proliferation in virtue of their cytological compatibility [70].

On the other side, modifications of the existing polyurethanes to improve their mechanical characteristics have been experimented. Addition of a gelatin-based hydrophilic sheath to the hydrophobic core of polyurethane determined significant changes in the core-sheath structures, and consequently in their mechanical properties, leading to the generation of scaffolds with tissue-like viscoelasticity, high compliance, competent tensile modulus and advantageous resistance to burst pressure, and suture retention [71]. However, the same authors demonstrated the superiority in terms of elastomechanical properties of bioresorbable polymers as polycaprolactone or polylactic acid, which added the benefit of an improved biocompatibility due to the nature of the material and to the nanofibrous extracellular matrix-like (ECM) architecture in which this polymer can be structured [71]. The principle of biomimesis of the nanoscale fibrillar features of the native ECM is crucial in vascular tissue engineering to achieve tissue-like mechanical and biological properties [67, 72, 73]. For example, the use of electrospinning technique, a manufacturing approach enabling the generation of scaffolds reproducing the native histoarchitecture of the fibrillar ECM [67, 74], has been shown to allow the realization of arterial surrogates with native-like mechanical properties [68, 75, 76]. However, several other tissue engineering methods have been developed for the fabrication of three-dimensional biomimetic scaffolds [73]. Despite that a complete examination of these approaches is out of the scope of this review, it is important to notice that the general tendency of vascular tissue engineering research is currently inspired by a biomimetic rationale and is focused on the realization of vascular surrogates able to closely simulate the natural biological and physical properties of native vessels and reproduce a similar biomechanical behavior when implanted in vivo settings. Future researches in this field may provide vascular scaffolds resembling native tissues to be used as aortic substitutes in routine clinical practice, overcoming the limitations associated with current Dacron grafts.

In this framework, computational fluid dynamic (CFD) studies are rapidly emerging in the cardiovascular field providing interesting insights on several hemodynamic aspects, involving vascular prostheses and allowing to evaluate performance and compare the effectiveness of aortic substitutes [77–79]. Blood flow, wall shear stress, and compliance can be accurately evaluated, and this method has been recently introduced to assess the hemodynamic effects of aortic surgery [79, 80]. CFD confirmed the importance of Valsalva sinuses in aortic root dynamics and established that stiffness of Dacron conduits still represents a main concern, despite that synthetic grafts share design and many hemodynamic parameters with the native aorta [81]. Even more interestingly, CFD studies demonstrated that also the surgical technique employed in the aortic replacement might play a role in determining the generation of unfavorable hemodynamic conditions, with consequent reflexes on the formation of aneurysms or pseudoaneurysms. Indeed, the reestablishment of normal aortic curvature, with a smooth transition between the conduit and native aorta, is crucial to reduce wall stress and potential turbulent retrograde and recirculating flow in the aortic arch [81, 82]. CFD may also be used to provide a tailored and patient-specific approach to aorta replacement as able to predict the biomechanical behavior of grafts and native aorta according to the typology of operation performed. As an example, Heim et al. were able to predict the potential hemodynamical implications of the extent of surgical replacement of the aorta and of the choice of the distal anastomosis site of the artificial conduit, establishing the superiority of hemi-arch replacement over isolated ascending aorta replacement in terms of residual stress on aortic tissues [83]. These findings might have enormous implications in the clinical practice as patient-specific CFD studies could potentially aid the decision on the surgical strategy to be adopted according to the different clinical scenarios. Also, scaffolds produced using tissue engineering approach may be evaluated in vivo using this method in order to individuate the most suitable and biomimicking substitute for aorta replacement.

## Conclusions

Far to be provocative, the conclusion of this literature review does not pretend to neglect the uncontested success of synthetic materials as aortic substitutes and the benefit still now provided to millions of patients around the world since their introduction by Dr. Voorhes and soon after Dr. DeBakey. However, early studies already warned about the potential complications above described and found their rationale in the compliance mismatch between the native aorta and the artificial grafts. The collection of more contemporary experimental and clinical evidences confirms these concerns and might trigger discussion on the long-term consequences of Dacron graft implantation when approaching the surgical workup of aortic root disease.

The reconsideration of this apparently neglected problem might on a side prompt the choice of other surgical strategies as the use of xenografts, while on the other might trigger future research activities towards the development alternative conduits mimicking the native biomechanical vascular properties. In this context, the new advances in tissue engineering of blood vessels using bioresorbable materials able to functionally integrate with the host tissue and induce a natural process of arterialization in vivo might hold a promise for the future. Would Leonardo Da Vinci, who firstly discovered the importance of aortic root dynamics, have imagined this?

## Abbreviations

ECM: Extracellular matrix
ePTFE: Polytetrafluoroethylene
PWV: Aortic pulsed wave velocity
